# Enhanced Dissolution of Naproxen by Combining Cocrystallization and Eutectic Formation

**DOI:** 10.3390/pharmaceutics13050618

**Published:** 2021-04-25

**Authors:** Hakyeong Kim, Soeun Jang, Il Won Kim

**Affiliations:** Department of Chemical Engineering, Soongsil University, Seoul 06978, Korea; hacc02@naver.com (H.K.); linda715@naver.com (S.J.)

**Keywords:** active pharmaceutical ingredient, naproxen, cocrystal, eutectic, dissolution

## Abstract

Improving dissolution properties of active pharmaceutical ingredients (APIs) is a critical step in drug development with the increasing occurrence of sparingly soluble APIs. Cocrystal formation is one of the methods to alter the physicochemical properties of APIs, but its dissolution behavior in biorelevant media has been scrutinized only in recent years. We investigated the combined strategy of cocrystallization and eutectic formation in this regard and utilized the cocrystal model system of naproxen and three pyridinecarboxamide isomers. Binary melting diagrams were constructed to discover the eutectic compositions of the three cocrystals with excess amounts of pyridinecarboxamides. The melt–crystallized eutectics and cocrystals were compared in their dissolution behaviors with respect to neat naproxen. The eutectics enhanced the early dissolution rates of the cocrystals in both the absence and presence of biologically relevant bile salt and phospholipid components, whereas the cocrystal dissolution was expedited and delayed, respectively. The combined strategy in the present study will be advantageous in maximizing the utility of the pharmaceutical cocrystals.

## 1. Introduction

About 70–90% of drug candidates suffer inadequate solubilities, presenting a major obstacle to overcome in drug developments [1,2]. When the solubility of an active pharmaceutical ingredient (API) is too low for the dose required for the intended efficacy (dose/solubility ratio > 250 mL), the API is categorized as class II or IV of the biopharmaceutical classification system (BCS) depending on its permeation characteristic: II for proper and IV for improper gastrointestinal permeability [3,4]. Since the oral bioavailability of the class II APIs is limited by their dissolution, improving their solubility and/or dissolution rate has been the special focus of many studies [3,4,5,6,7,8,9].

Improvement of API dissolution behavior has been attempted from the perspectives of kinetics and thermodynamics, both of which can be easily explained using the classic Noyes–Whitney equation, where the dissolution rate is proportional to the surface area and solubility [10]. A common kinetic approach is increasing the surface area to be in contact with fluids through, for example, nano–drug formation [5,6,7,8]. Enhancing the API solubility by altering the nature of the solids is the thermodynamic approach, employing salts, metastable polymorphs, amorphous phases, and cocrystals [4,7,8,11]. Among these solids, cocrystals take a unique place due to the diverse possibilities for the coformer selection and the stability arising from the strong hydrogen bonding often found in the cocrystal structures [9,11,12].

The cocrystal solubilities have been the subject of in–depth studies due to the complexity that originates from the presence of the coformers as well as the diverse in vivo environments [9,13,14]. Especially noteworthy are the cocrystal solubilities defined in both simple buffers and lipid–based media, which are physiologically more relevant [14,15,16]. The significant solubility difference in these environments clearly indicates the need for a more cautious interpretation of the improved solubilities of API cocrystals in simple buffers, which are the predominant choices in many previous studies [17,18].

**Figure 1:**
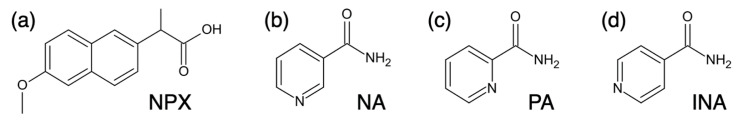
Molecular structures of (**a**) naproxen (NPX), (**b**) nicotinamide (NA), (**c**) picolinamide (PA), and (**d**) isonicotinamide (INA).

In the present study, we used a classic cocrystal system of naproxen (NPX) and pyridinecarboxamide coformers (nicotinamide (NA), pyridine–3–carboxamide; isonicotinamide (INA), pyridine–4–carboxamide; picolinamide (PA), pyridine–2–carboxamide) since the isomer coformers would allow a systematic investigation with minimal variations in chemistry (Figure 1) [19,20,21]. These cocrystals (NPX/NA = 2:1; NPX/INA = 1:1; NPX/PA = 1:1) of NPX, a class II API, have been investigated in several studies, but their in vitro dissolution behaviors were mostly in simple buffers [20,21,22]. We chose a fed state simulated intestinal fluid (FeSSIF) and a matching pH 5.0 buffer as the dissolution environments because they represent the condition recommended for the intake of naproxen—with food or milk to decrease gastrointestinal adverse effects [23]. These two conditions can be also considered as two extreme limits in the concentration range of the physiologically relevant surfactants, which can be in principle diverse under in vivo conditions [15,24]. In addition, since the layered nano–structures intrinsic to the eutectic solidification could increase the surface area during dissolution, the eutectic formation was attempted to improve the dissolution behavior of the cocrystals further after constructing the binary phase diagrams of naproxen and the coformers [25,26,27].

## 2. Materials and Methods

### 2.1. Materials

The active pharmaceutical ingredient (API) and three coformers were purchased from Sigma–Aldrich (St. Louis, MO, USA): S–naproxen (NPX: ≥98.5%, USP testing specifications), nicotinamide (NA: ≥99.5%), isonicotinamide (INA: 99%), and picolinamide (PA: 98%). Ethanol (anhydrous, 99.9%) was from Samchun Chemical (Seoul, Korea).

Dissolution media were a fed state simulated intestinal fluid (FeSSIF) and a pH 5.0 buffer solution. FaSSIF/FeSSIF/FaSSGF powder was obtained from Biorelevant (London, UK). Sodium chloride (NaCl, ≥99.5%), sodium hydroxide (NaOH, ≥97%), and acetic acid (CH_3_COOH, ≥99%) were from Sigma–Aldrich. Deionized (DI) water with a resistivity over 18.2 MΩ·cm was supplied from a Direct–Q3 water purification system (Millipore, Burlington, MA, USA). Lastly, 1 M HCl (aq) and 1 M NaOH (aq) were obtained from Samchun Chemical (Seoul, Korea) and Daejung Chemical (Gyeonggi, Korea), respectively.

### 2.2. Analysis of API/Coformer Mixtures with Cocrystal Formation

The mixtures of API (NPX) and coformers (NA, INA, or PA) were investigated using samples ball–milled with a Retsch MM 200 (Haan, Germany) to ensure the API/coformer cocrystallization. First, the API and coformers were individually powdered with an agate mortar and pestle for 2 min. Then, mixtures (100 mg scale) at appropriate API/coformer ratios were milled (30 min, 15 Hz) with 10 μL ethanol (typically 1 μL ethanol/10 mg solid mixture) in a cylindrical stainless–steel jar (diameter ~25 mm; length ~50 mm) containing two stainless steel balls (diameter ~9 mm).

The ball–milled mixtures were analyzed using a differential scanning calorimeter (DSC: DSC3 STARe system, Mettler–Toledo, Columbus, OH, USA) pre–calibrated for temperature and enthalpy using indium and zinc. DSC was performed under a nitrogen gas atmosphere, and the heating rate was 10 °C/min.

The mixtures were also analyzed by X–ray diffraction (XRD). A D2 PHASER diffractometer (Bruker AXS, Billerica, MA, USA) was operated in the *θ*–*θ* mode with CuKα radiation (*λ* = 1.5406 Å) at 30 kV and 10 mA. A zero–background sample holder (Bruker AXS) was used for better sensitivity, and the diffraction data were collected in the 2*θ* range of 5–35° at 0.02° increments (scanning rate 1°/min).

### 2.3. Characterization of Melt Crystallized Mixtures

Some mixtures were further melt crystallized after the ball–milling process. Ball–milled samples were melted in aluminum dishes on a 170 °C hot plate (AREX–6, VELP Scientifica, Usmate Velate, Italy), and they were immediately moved and crystallized in a 25 °C incubator for 24 h (BF–150LI, BioFree, Seoul, Korea). The melt crystallized samples were lightly ground with an agate mortar and pestle for 2 min before further characterization. The structures of the melt crystallized samples were analyzed by XRD as described in the previous section.

In vitro release behaviors of NPX from the melt crystallized mixtures were studied at 37 °C using a USP type II apparatus (paddle) at 100 rpm (RC–3 dissolution tester, Minhua Pharmaceutical Machinery, Shanghai, China). Dissolution media were FeSSIF (pH 5.0) and a pH 5.0 buffer solution, and a volume of 500 mL was used with the appropriate amount of mixtures to contain 320 mg NPX. Dissolution was monitored at 5, 10, 15, 20, 30, 40, 60, 90, and 120 min by withdrawing 3–mL aliquots of the solution and adding an equal amount of the fresh solution to maintain a constant volume. Each aliquot was filtered through a 0.20–μm PTFE filter (Advantec, Tokyo, Japan), and its NPX concentration was measured through UV absorbance (V730, Jasco, Tokyo, Japan) at 331.8 nm (FeSSIF) or 330.6 nm (pH 5.0 buffer), where the absorbance of the coformers was absent. Additionally, the remaining solids after the dissolution at non–sink conditions were analyzed using XRD. FeSSIF was prepared as instructed in the recipe of the supplier (Biorelevant, London, UK), and it contained sodium taurocholate (15 mM), lecithin (3.75 mM), sodium chloride (203 mM), sodium hydroxide (101 mM), and acetic acid (144 mM). The pH 5.0 buffer solution was the same except that sodium taurocholate and lecithin were absent. All dissolution experiments were repeated in triplicate.

The melt crystallization behavior was also studied using an optical microscope (OM: BX–51, Olympus, Tokyo, Japan). The solid samples were melted using a hot stage (FP90, Mettler–Toledo, Columbus, OH, USA), and their crystallization behavior during cooling was monitored under cross polarization with the first–order retardation plate.

## 3. Results and Discussion

### 3.1. Melting Behaviors of Naproxen and Pyridinecarboxamide Coformers

**Figure 2:**
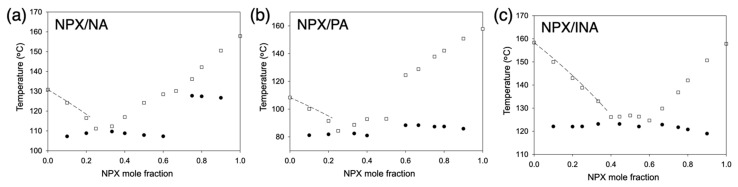
Melting diagrams of (**a**) NPX/NA, (**b**) NPX/PA, and (**c**) NPX/INA mixtures (differential scanning calorimeter (DSC) heating rate 10 °C/min). Dashed lines indicate ideal behaviors calculated with the van’t Hoff equation; empty squares indicate liquidus temperatures; filled circles indicate solidus temperatures.

**Figure 3:**
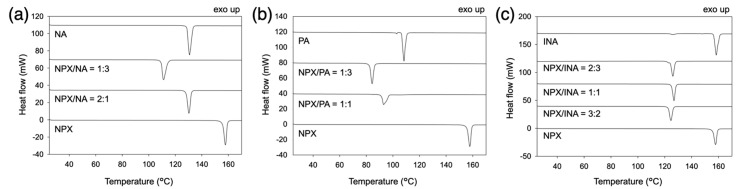
DSC thermograms of some mixtures of (**a**) NPX/NA, (**b**) NPX/PA, and (**c**) NPX/INA.

Melting behaviors of naproxen (NPX) and pyridinecarboxamide coformers (nicotinamide, NA; picolinamide, PA; isonicotinamide, INA) are shown in Figure 2. The solidus boundaries (marked with filled circles) are obtained as the onsets of the first endothermic peaks of DSC thermograms. The liquidus boundaries (empty squares) are measured as the peak points of the final melting endotherms since some peaks were partly overlapped or quite broad. All DSC thermograms for the construction of melting diagrams are shown in Figure S1, and those for cocrystals and eutectic compositions are also presented in Figure 3 along with the neat compounds. Note that the samples were subject to the liquid–assisted grinding before DSC measurements to induce cocrystal formation between naproxen and coformers, and the successful cocrystallization was confirmed via XRD, as shown in Figure S2.

The compositions of the cocrystals were in agreement with the previously reported structures (Figure 2) [19,20,21]: NPX/NA = 2:1, NPX/PA = 1:1, and NPX/INA = 1:1. Those of the eutectics were NPX/NA = 1:3, NPX/PA = 1:3, and NPX/INA = 2:3 and 3:2. Therefore, all eutectics are between the cocrystals and the corresponding coformers except NPX/INA = 3:2. The cocrystals and eutectics display single melting endotherms in their DSC thermograms (Figure 3). (We note that more precise eutectic compositions could be obtained with a much lower heating rate of DSC.) Additionally, small endotherms related to the polymorphism of PA and INA are visible in the thermograms of neat PA, neat INA, and NPX/INA = 2:3 [28,29,30].

At eutectic compositions, the participating species melt simultaneously, and the complete liquid state is obtained at the lowest temperature (Figure 2). When the liquid is solidified by cooling, the individual species are crystallized separately to form lamellar structures where each crystal species is surrounded by the other component [26,27,31,32]. Since finely divided structures of APIs could be helpful to enhance their dissolution rate, the eutectic formation of sparingly soluble APIs has attracted significant attention [25,27,31,33,34,35]. Still, not all pairs of materials form eutectics. Favorable molecular interactions, as well as similar melting points, are the suggested requirements for the successful eutectic formation [25,31,36,37,38]. For example, naproxen did not form eutectics with fatty alcohols, such as octadecanol and docosanol, whereas ibuprofen with a similar molecular structure successfully formed eutectics [38]. This is one of the reasons why we explored the cocrystals of naproxen since cocrystals are likely interacting favorably with an excess amount of its constituting components to make eutectic formation successful [18,39,40].

Melting point depressions of the pyridinecarboxamides were analyzed using the van’t Hoff equation to assess the intermolecular interactions in the liquid phase [41]:

$$\frac{1}{T_{fus}}=\frac{1}{T_{fus,coformer*}}–\frac{R}{\Delta {Η}_{fus,coformer*}}\ln\left(\gamma_{coformer}\;x_{coformer}\right)$$

where *x_coformer_*, *γ_coformer_*, and *R* are the mole fraction of a pyridinecarboxamide coformer, activity coefficient, and gas constant, respectively; ΔH_fus,coformer*_ and T_fus,coformer*_ are the molar enthalpy of fusion and melting point of the pure coformer, respectively; *T*_*fus*_ is the melting point at *x_coformer_*. The dashed lines in Figure 2 represent the ideal behaviors in the pyridinecarboxamide–rich regions, where the activity coefficient in the liquid solution (*γ*_NA_, *γ*_PA_, or *γ*_INA_) is unity. The actual melting point depressions were not far away from the ideal behaviors (e.g., when *x*_NPX_ = 0.2, *γ*_NA_ = 0.97, *γ*_PA_ = 0.94, and *γ*_INA_ = 0.98), which did not suggest particularly strong interactions. This could be because all NPX molecules were already strongly interacting with the pairing pyridinecarboxamide molecules in the liquid solutions, and the excess amount of the NA, PA, or INA could not form additional intermolecular interactions strongly.

An alternative simple approach to predict the eutectic formation of the cocrystals derived from van’t Hoff equation is using an index called *I*_c_, which essentially relies on the closeness in melting points [36,38]:

$$I_{c}=\frac{\Delta H_{fus,cocrystal}}{R}\left(\frac{1}{T_{fus,excess}}-\frac{1}{T_{fus,cocrystal}}\right)$$

where *T_fus,excess_* is the melting point of the excess species, and *T_fus,cocrystal_* and Δ*H_fus,cocrystal_* are the melting point and the molar enthalpy of fusion of a cocrystal, respectively.

When the excess species was the corresponding pyridinecarboxamide of each cocrystal, |*I*_c_| was calculated as 0.04, 0.47, and 1.02 for NPX/NA, NPX/PA, and NPX/INA cocrystals, respectively. When it was NPX, |*I*_c_| was 1.50, 1.77, and 1.00 for NPX/NA, NPX/PA, and NPX/INA cocrystals, respectively. This approach is in good agreement with the experimental results of the melting diagrams since smaller *I*_c_ values indicate a higher possibility of eutectic formation [36]. Since closeness in melting points is a natural requirement of co–solidification, it appears that the pyridinecarboxamides, especially NA and PA, could form eutectics with the corresponding cocrystals even without strong deviations from the ideal behavior of the mixtures.

### 3.2. Melt Crystallization of the Cocrystals

Melt recrystallization of the cocrystals was performed after liquid–assisted grinding to erase any distortions involved with the mechanically severe milling conditions, and it was also a required process for the eutectic compositions to form actual eutectic structures [32,33,34,35,38]. After complete melting, the melt was crystallized in a 25 °C incubator for 24 h, and the crystallized samples were examined with DSC (Figure S3). Both NPX/NA and NPX/PA cocrystals showed the melting enthalpy comparable to that of milled samples (ca. 134–135 J/g for NPX/NA and 97–102 J/g for NPX/PA), indicating complete recrystallization. No additional crystallization exotherm existed during DSC scanning. In contrast, NPX/INA cocrystal displayed a crystallization exotherm before the melting endotherm, reflecting incomplete melt recrystallization.

**Figure 4:**
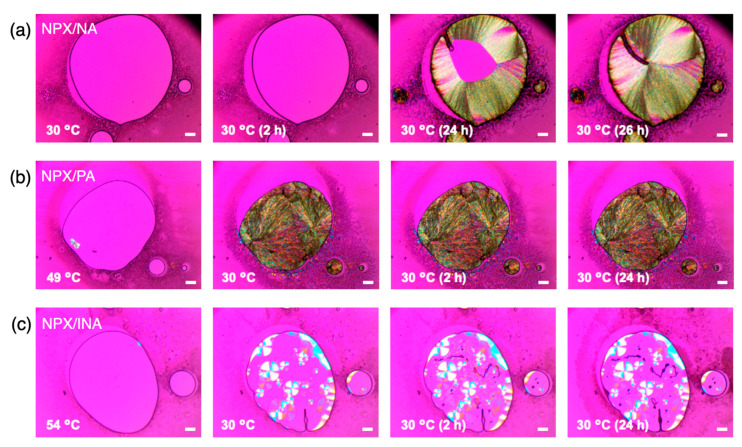
Optical microscope (OM) micrographs during melt crystallization of the cocrystals of (**a**) NPX/NA (2:1), (**b**) NPX/PA (1:1), and (**c**) NPX/INA (1:1). The observation was under cross polarization, and all scale bars are 100 μm. Note that the crystallization of NPX/PA and NPX/INA cocrystals started during cooling at about 49 °C and 54 °C, respectively.

The delayed crystallization of NPX/INA was also confirmed with optical microscopy coupled with a hot stage. Figure 4 shows the micrographs observed under cross–polarization during cooling of the melt (a: NPX/NA; b: NPX/PA; c: NPX/INA). (Note that the isotherm of the melt crystallization was at 30 °C in this case for the stable temperature control of the hot stage.) After cooling to 30 °C and subsequent crystallization for 24 h, an amorphous phase of NPX/INA without birefringence existed in a large portion (Figure 4c), and it was sustained even longer without further change. Interestingly, the initially formed crystal spherulites did not propagate further to increase their size, and the remaining regions were filled with the mixture of weakly birefringent and apparently amorphous materials with seemingly no advancement of crystallization. In contrast, near–complete crystallization was observed for NPX/NA (Figure 4a) and NPX/PA (Figure 4b), and particularly fast recrystallization was found for NPX/PA.

The kinetics of melt crystallization is known to be balanced by the sufficient driving force of the supercooling and the proper fluidity away from the glass transition [42]. Melting points of the NPX/NA, NPX/PA, and NPX/INA are about 130 °C, 93 °C, and 127 °C, respectively. If the glass transition temperature (*T*_g_) is estimated as 2/3 of *T*_m_ (in K), they are about −4 °C, −29 °C, and −6 °C for NPX/NA, NPX/PA, and NPX/INA, respectively [42]. Therefore, the fast crystallization of NPX/PA could be attributed to its relatively low glass transition temperature, the ambient temperature being approximately midpoint between Tm and *T*_g_. However, the kinetic difference between NPX/NA and NPX/INA seems to arise from the variations in the molecular interactions, which is globally expressed as the different molar ratio of the cocrystals (NPX/NA = 2:1 and NPX/INA = 1:1) and locally indicated by the different synthons observed in the cocrystal structures [19,20,21]. Further study would be necessary to understand the exact nature of the kinetics of the melt crystallization of the cocrystals.

Overall, the melt crystallization of NPX/NA and NPX/PA after liquid–assisted grinding was successfully performed, eliminating any milling–related histories. NPX/INA, on the other hand, showed incomplete melt crystallization behavior. Therefore, the dissolution behaviors of the cocrystals and their eutectic compositions were studied only for NPX/NA and NPX/PA, as described in the following section.

### 3.3. In Vitro Release Behaviors of the Cocrystals and Eutectics

**Figure 5:**
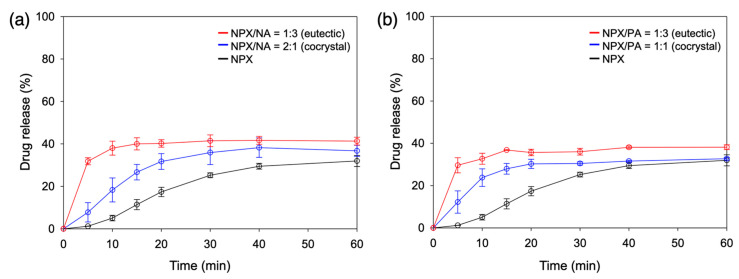
Dissolution profiles (pH 5.0, 60 min, *n* = 3) of (**a**) NPX/NA and (**b**) NPX/PA. Eutectics and cocrystals are compared with neat NPX.

Since it is recommended to take naproxen with food, the FeSSIF and the corresponding pH 5.0 buffer media were employed to assess the dissolution behaviors of NPX/NA and NPX/PA cocrystals and eutectics [23]. Figure 5 shows their dissolution profiles in the pH 5.0 buffer. Both NPX/NA and NPX/PA cocrystals showed clearly improved dissolution behaviors, especially at the early stage (<30 min). For example, the NPX release after 10 min was 5.0 ± 1.3%, 18.3 ± 5.6%, and 23.8 ± 4.1% for neat NPX, NPX/NA cocrystal, and NPX/PA cocrystal, respectively. This can be explained as the aqueous environments easily disrupting the structurally integral hydrogen bonding of the cocrystals [20,21]. The NPX release was further accelerated when the eutectic compositions were utilized: 38.0 ± 3.3% and 32.7 ± 2.6% after 10 min for the eutectics of NPX/NA and NPX/PA, respectively. Eutectic structures with finely divided cocrystals appear to be the main cause of boosting the early dissolution of NPX [25,27,31,38].

**Figure 6:**
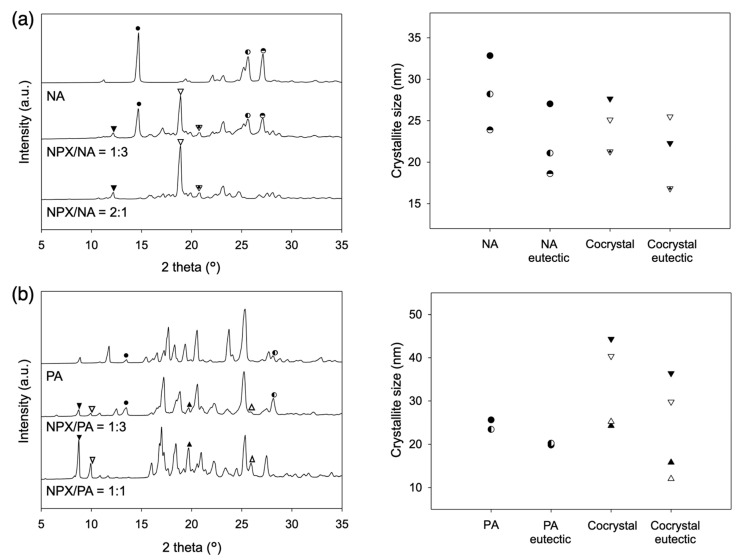
XRD patterns of the eutectics compared with neat cocrystals/coformers and the calculated crystallite size: (**a**) NPX/NA; (**b**) NPX/PA.

The microstructures of the eutectics of NPX/NA and NPX/PA were analyzed using the Scherrer equation:

$$L=\frac{K\lambda }{\beta cos\theta }$$

where crystallite size *L* is calculated as a function of the full width at half maximum (*β*) of the diffraction peak at angle 2*θ* with the X–ray wavelength *λ* (1.5406 Å) and constant *K* (shape factor commonly taken as 0.9) [43]. Figure 6 shows the XRD patterns and the results of the analysis, indicating a substantial reduction of the crystallite size with the eutectic structures. Diffraction peaks distinctive for the cocrystal phases were marked with the triangles and those for the excess pyridinecarboxamides were marked with the circles. The crystallite size decreased with the eutectic formation by average 12.9%, 21.7%, 32.8%, and 18.1% for the NPX/NA cocrystal, NA, NPX/PA cocrystal, and PA, respectively. Note that the reduction of the crystallite size per se would be only partially responsible for the accelerated dissolution of the eutectics since the crystallites of the eutectics could distribute as the fine grains within the characteristic lamellar structures, whereas those of the stoichiometric cocrystals should form denser and larger particles.

When additional coformers (NA or PA) were simply mixed with the cocrystals to make eutectic compositions (NPX/NA or NPX/PA = 1:3) without actually achieving eutectic structures, the release rate was faster than that of neat cocrystals but slower than that of eutectics (29.6 ± 2.0% for NPX/NA and 28.9 ± 1.9% for NPX/PA after 10 min, data not shown), which demonstrated some complexation effect of the coformers once dissolved [9,13]. Note that the remaining solids after the dissolution study were neat NPX irrespective of the starting materials, which indicated that the dissolution of the cocrystals was eventually limited by the recrystallization of the less soluble NPX phase. This also explains the relative convergence of the amount of drug release after 120 min, as shown in Figure S4a (ca. 33.9 ± 2.5% neat NPX; 40.0 ± 4.8% NPX/NA cocrystal; 42.2 ± 2.1% NPX/NA eutectic; 31.6 ± 0.9% NPX/PA cocrystal; 38.5 ± 1.6% NPX/PA eutectic). The modest differences in the final release percentage appear to arise from the varied ability of the coformers acting as solubilizing agents. The p*K*_a_ values of the conjugated acids of the pyridine part of NA and PA are estimated as 3.63 and 1.17, respectively, and the less basic nature of PA is attributed to its ortho structure (Figure 1) [21]. This explains the lack of the hydrogen bonding of the pyridine part of PA to the NPX in the cocrystal structure of NPX/PA, and it is also consistent with the smaller melting enthalpy of NPX/PA (ca. 100 J/g compared to ca. 130 J/g for NPX/NA) observed in the current study [21]. Therefore, it is not unreasonable to assume that NA acts as a better solubilizer with stronger interactions with NPX than PA does. The main conclusion of the dissolution study in the pH 5.0 buffer is that the NPX/NA and NPX/PA cocrystals are both effective in increasing the early release of NPX, and their eutectics more so.

**Figure 7:**
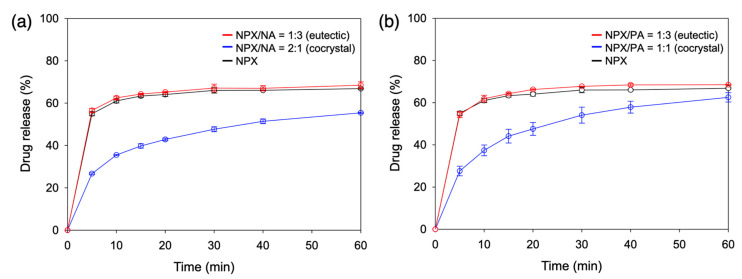
Dissolution profiles (fed state simulated intestinal fluid (FeSSIF), 60 min, *n* = 3) of (**a**) NPX/NA and (**b**) NPX/PA. Eutectics and cocrystals are compared with neat NPX.

Figure 7 and Figure S4b show the dissolution behaviors of the NPX cocrystals and eutectics in FeSSIF. For all starting materials, dissolution was expedited compared to that in the pH 5.0 buffer. However, the extent of the dissolution acceleration was different enough to alter the order of the early dissolution rate: eutectics ~ neat NPX > cocrystals (whereas eutectics > cocrystals > neat NPX in the pH 5.0 buffer). The dramatic increase of the early dissolution of neat NPX (e.g., after 10 min 61.0 ± 1.0% in FeSSIF vs. 5.0 ± 1.3% in pH 5.0 buffer) indicates that the bile salt and phospholipid components in FeSSIF were quite effective in solubilizing the hydrophobic NPX molecules. This is in good agreement with the fact that the colloidal assemblies of the bile salt and phospholipid components, found in both the simulated and human intestinal fluids, are playing significant roles in increasing the solubilization of hydrophobic APIs [15,44,45,46,47].

In contrast, the acceleration (pH 5.0 vs. FeSSIF) was substantial but not as extensive for the cocrystal and their eutectics (e.g., after 10 min 38.0 ± 3.3% to 62.5 ± 0.8% for NPX/NA eutectic; 18.3 ± 5.6% to 35.5 ± 0.3% for NPX/NA cocrystal; 32.7 ± 2.6% to 61.9 ± 1.6% for NPX/PA eutectic; 23.8 ± 4.1% to 37.4 ± 2.6% for NPX/PA cocrystal). We presume that the NPX molecules interacting with the hydrophilic pyridinecarboxamides (NA and PA) are less susceptible to the solubilizing actions of the bile salt and lipid additives in FeSSIF. Similar behaviors are seen with a variety of solubilizing agents, and they are explained based on the preferential solubilization of the hydrophobic APIs over the coformers, where the API solubility exceeds cocrystal solubility above certain critical concentrations of the solubilizers [14,15]. The dissolution behaviors of the NPX cocrystals in FeSSIF appear to be governed by similar phenomena, whereas those of the eutectics are probably compensated by the finely divided eutectic structures. Again, the remaining solid after dissolution in FeSSIF was neat NPX in all cases, which explains the relatively smaller difference of dissolution percentage after 120 min, as shown in Figure S4b (ca. 67.3 ± 0.9% neat NPX; 58.8 ± 3.1% NPX/NA cocrystal; 67.6 ± 0.5% NPX/NA eutectic; 69.2 ± 0.5% NPX/PA cocrystal; 68.6 ± 0.3% NPX/PA eutectic). The main conclusion of the dissolution study in FeSSIF is that both NPX/NA and NPX/PA cocrystals are surprisingly ineffective in increasing the early release of NPX, and their eutectics less so.

Overall, accelerated early dissolution in both pH 5.0 buffer and FeSSIF was observed for the eutectics of NPX cocrystals and excess coformers in comparison to NPX or simple cocrystals. The favorable dissolution behavior of the eutectics could be attributed to their finely divided structures, especially because the simple cocrystals with hydrophilic coformers exhibited the slowest dissolution in FeSSIF.

## 4. Conclusions

In summary, the melting behaviors of NPX and three pyridinecarboxamide isomers were studied under the conditions that favored the cocrystallization of NPX/pyridinecarboxamide. All three cocrystals showed eutectic compositions with appropriate amounts of pyridinecarboxamides. NPX/NA and NPX/PA, which displayed proper melt crystallization, were further investigated in regard to their dissolution behaviors. The eutectics enhanced the dissolution rate of the cocrystals in both conditions, where the cocrystal dissolution was expedited (pH 5.0 buffer) and delayed (FeSSIF), which indicated that the layered micro– and nano–structures of the eutectics were mainly responsible for the enhancement. The current study demonstrated the utility of eutectics to improve the dissolution properties of the cocrystals. Further studies with varied hydrophobicity and hydrophilicity of APIs and coformers would be necessary to firmly establish the protocol to maximize the dissolution properties of the cocrystals using eutectics.

## Data Availability

Not applicable.

## Supplementary Materials

The following are available online at https://www.mdpi.com/article/10.3390/pharmaceutics13050618/s1. Figure S1: DSC thermograms of (a) NPX/NA, (b) NPX/PA, and (c) NPX/INA mixtures; Figure S2: XRD patterns of some mixtures of (a) NPX/NA, (b) NPX/PA, and (c) NPX/INA mixtures; Figure S3: DSC thermograms of melt crystallized cocrystals of (a) NPX/NA, (b) NPX/PA, and (c) NPX/INA; Figure S4: Full dissolution profiles (120 min, *n* = 3) of NPX/NA and NPX/PA in (a) pH 5.0 buffer and (b) FeSSIF.

## References

[1] Kawabata Y., Wada K., Nakatani M., Yamada S., Onoue S. (2011). Formulation design for poorly water-soluble drugs based on biopharmaceutics classification system: Basic approaches and practical applications. Int. J. Pharm..

[2] Loftsson T., Brewster M.E. (2010). Pharmaceutical applications of cyclodextrins: Basic science and product development. J. Pharm. Pharmacol..

[3] Florence A.T., Attwood D. (2016). Physicochemical Principles of Pharmacy: In Manufacture, Formulation and Clinical Use.

[4] He X., Qiu Y., Chen Y., Zhang G.G.Z., Liu L., Porter W.R. (2009). Integration of physical, chemical, mechanical, and biopharmaceutical properties in solid oral dosage form development. Developing Solid Oral Dosage Forms.

[5] Kesisoglou F., Panmai S., Wu Y. (2007). Nanosizing—Oral formulation development and biopharmaceutical evaluation. Adv. Drug. Deliv. Rev..

[6] Shegokar R., Müller R.H. (2010). Nanocrystals: Industrially feasible multifunctional formulation technology for poorly soluble actives. Int. J. Pharm..

[7] Jermain S.V., Brough C., Williams III R.O. (2018). Amorphous solid dispersions and nanocrystal technologies for poorly water-soluble drug delivery—An update. Int. J. Pharm..

[8] Boyd B.J., Bergström C.A.S., Vinarov Z., Kuentz M., Brouwers J., Augustijns P., Brandl M., Bernkop-Schnürch A., Shrestha N., Préat V. (2019). Successful oral delivery of poorly water-soluble drugs both depends on the intraluminal behavior of drugs and of appropriate advanced drug delivery systems. Eur. J. Pharm. Sci..

[9] Rodríguez-Hornedo N., Nehm S.J., Jayasankar A., Swarbrick J. (2007). Cocrystals: Design, properties and formation mechanisms. Encyclopedia of Pharmaceutical Technology.

[10] Noyes A.A., Whitney W.R. (1897). The rate of solution of solid substances in their own solution. J. Am. Chem. Soc..

[11] Jones W., Motherwell W.D.S., Trask A.V. (2006). Pharmaceutical cocrystals: An emerging approach to physical property enhancement. MRS Bull..

[12] Wood P.A., Feeder N., Furlow M., Galek P.T.A., Groom C.R., Pidcock E. (2014). Knowledge-based approaches to co-crystal design. CrystEngComm.

[13] Rao V.M., Sanghvi R., Zhu H., Qiu Y., Chen Y., Zhang G.G.Z., Liu L., Porter W.R. (2009). Solubility of pharmaceutical solids. Developing Solid Oral Dosage Forms.

[14] Lipert M.P., Rodríguez-Hornedo N. (2015). Cocrystal transition points: Role of cocrystal solubility, drug solubility, and solubilizing agents. Mol. Pharm..

[15] Lipert M.P., Roy L., Childs S.L., Rodríguez-Hornedo N. (2015). Cocrystal solubilization in biorelevant media and its prediction from drug solubilization. J. Pharm. Sci..

[16] Machado T.C., Kuminek G., Cardoso S.G., Rodríguez-Hornedo N. (2020). The role of pH and dose/solubility ratio on cocrystal dissolution, drug supersaturation and precipitation. Eur. J. Pharm. Sci..

[17] Thakuria R., Delori A., Jones W., Lipert M.P., Roy L., Rodríguez-Hornedo N. (2013). Pharmaceutical cocrystals and poorly soluble drugs. Int. J. Pharm..

[18] Évora A.O.L., Castro R.A.E., Maria T.M.R., Silva M.R., ter Horst J.H., Canotilho J., Eusébio M.E.S. (2014). A thermodynamic based approach on the investigation of a diflunisal pharmaceutical co-crystal with improved intrinsic dissolution rate. Int. J. Pharm..

[19] Castro R.A.E., Ribeiro J.D.B., Maria T.M.R., Silva M.R., Yuste-Vivas C., Canotilho J., Eusébio M.E.S. (2011). Naproxen cocrystals with pyridinecarboxamide isomers. Cryst. Growth Des..

[20] Ando S., Kikuchi J., Fujimura Y., Ida Y., Higashi K., Moribe K., Yamamoto K. (2012). Physicochemical characterization and structural evaluation of a specific 2:1 cocrystal of naproxen–nicotinamide. J. Pharm. Sci..

[21] Kerr H.E., Softley L.K., Suresh K., Hodgkinson P., Evans I.R. (2017). Structure and physicochemical characterization of a naproxen–picolinamide cocrystal. Acta Cryst..

[22] Abbas N., Latif S., Afzal H., Arshad M.S., Hussain A., Sadeeqa S., Bukhari N.I. (2018). Simultaneously improving mechanical, formulation, and in vivo performance of naproxen by co-crystallization. AAPS PharmSciTech.

[23] Lacy C.F., Armstrong L.L., Goldman M.P., Lance L.L. (2008). Drug Information Handbook: A Comprehensive Resource for All Clinicians and Healthcare Professionals.

[24] Galia E., Nicolaides E., Hörter D., Löbenberg R., Reppas C., Dressman J.B. (1998). Evaluation of various dissolution media for predicting in vivo performance of class I and II drugs. Pharm. Res..

[25] Cherukuvada S., Nangia A. (2014). Eutectics as improved pharmaceutical materials: Design, properties and characterization. Chem. Commun..

[26] Jain H., Khomane K.S., Bansal A.K. (2014). Implication of microstructure on the mechanical behaviour of an aspirin–paracetamol eutectic mixture. CrystEngComm.

[27] Figueirêdo C.B.M., Nadvorny D., de Medeiros Vieira A.C.Q., Sobrinho J.L.S., Neto P.J.R., Lee P.I., de La Roca Soares M.F. (2017). Enhancement of dissolution rate through eutectic mixture and solid solution of posaconazole and benznidazole. Int. J. Pharm..

[28] Skorupska E., Jeziorna A., Potrzebowski M.J. (2016). Thermal solvent-free method of loading of pharmaceutical cocrystals into the pores of silica particles: A case of naproxen/picolinamide cocrystal. J. Phys. Chem. C.

[29] Évora A.O.L., Castro R.A.E., Maria T.M.R., Rosado M.T.S., Silva M.R., Canotilho J., Eusébio M.E.S. (2012). Resolved structures of two picolinamide polymorphs. Investigation of the dimorphic system behaviour under conditions relevant to co-crystal synthesis. CrystEngComm.

[30] Li J., Bourne S.A., Caira M.R. (2011). New polymorphs of isonicotinamide and nicotinamide. Chem. Commun..

[31] Law D., Wang W., Schmitt E.A., Qiu Y., Krill S.L., Fort J.J. (2003). Properties of rapidly dissolving eutectic mixtures of poly(ethylene glycol) and fenofibrate: The eutectic microstructure. J. Pharm. Sci..

[32] Callister W.D., Rethwisch D.G. (2014). Materials Science and Engineering: An Introduction.

[33] Sekiguchi K., Obi N. (1961). Studies on absorption of eutectic mixture. I. A comparison of the behavior of eutectic mixture of sulfathiazole and that of ordinary sulfathiazole in man. Chem. Pharm. Bull..

[34] Goldberg A.H., Gibaldi M., Kanig J.L. (1966). Increasing dissolution rates and gastrointestinal absorption of drugs via solid solutions and eutectic mixtures. III. Experimental evaluations of griseofulvin–succinic acid solid solution. J. Pharm. Sci..

[35] Chiou W.L., Niazi S. (1971). Phase diagram and dissolution-rate studies on sulfathiazole–urea solid dispersions. J. Pharm. Sci..

[36] Law D., Wang W., Schmitt E.A., Long M.A. (2002). Prediction of poly(ethylene glycol)-drug eutectic compositions using an index based on the van’t Hoff equation. Pharm. Res..

[37] Vippagunta S.R., Wang Z., Hornung S., Krill S.L. (2007). Factors affecting the formation of eutectic solid dispersions and their dissolution behavior. J. Pharm. Sci..

[38] Jin S., Jang J., Lee S., Kim I.W. (2020). Binary mixtures of some active pharmaceutical ingredients with fatty alcohols—the criteria of successful eutectic formation and dissolution improvement. Pharmaceutics.

[39] Machado S.M.T., Castro R.A.E., Maria T.M.R., Canotilho J., Eusébio M.E.S. (2017). Levetiracetam + nonsteroidal anti-inflammatory drug binary systems: A contribution to the development of new solid dosage forms. Int. J. Pharm..

[40] An H., Choi I., Kim I.W. (2019). Melting diagrams of adefovir dipivoxil and dicarboxylic acid: An approach to assess cocrystal compositions. Crystals.

[41] Levine I.N. (2009). Physical Chemistry.

[42] Sperling L.H. (1993). Introduction to Physical Polymer Science.

[43] Cullity B.D., Stock S.R. (2014). Elements of X-ray Diffraction.

[44] Elvang P.A., Hinna A.H., Brouwers J., Hens B., Augustijns P., Brandl M. (2016). Bile salt micelles and phospholipid vesicles present in simulated and human intestinal fluids: Structural analysis by flow field–flow fractionation/multiangle laser light scattering. J. Pharm. Sci..

[45] Elvang P.A., Stein P.C., Brauer-Brandl A., Brandl M. (2017). Characterization of co-existing colloidal structures in fasted state simulated fluids FaSSIF: A comparative study using AF4/MALLS, DLS and DOSY. J. Pharm. Biomed. Anal..

[46] Clulow A.J., Parrow A., Hawley A., Khan J., Pham A.C., Larsson P., Bergström C.A.S., Boyd B.J. (2017). Characterization of solubilizing nanoaggregates present in different versions of simulated intestinal fluid. J. Phys. Chem. B.

[47] Jara M.O., Warnken Z.N., Williams R.O. (2020). Amorphous solid dispersions and the contribution of nanoparticles to in vitro dissolution and in vivo testing: Niclosamide as a case study. Pharmaceutics.

