# Supplementary material for: Enhanced Dissolution of Naproxen by Combining Cocrystallization and Eutectic Formation
Source: Pharmaceutics. 2021 Apr 25;13(5):618. doi: 10.3390/pharmaceutics13050618 (PMC8145234; doi:10.3390/pharmaceutics13050618)
Supplement: Supplementary file 1 [file pharmaceutics-13-00618-s001.zip › pharmaceutics-1169750-supplementary.pdf]

pharmaceutics

MDPI

Supplementary Materials: Enhanced Dissolution of Naproxen by Combining Cocrystal-li-zation and Eutectic Formation

Hak Yeong Kim, Soeun Jang and Il Won Kim

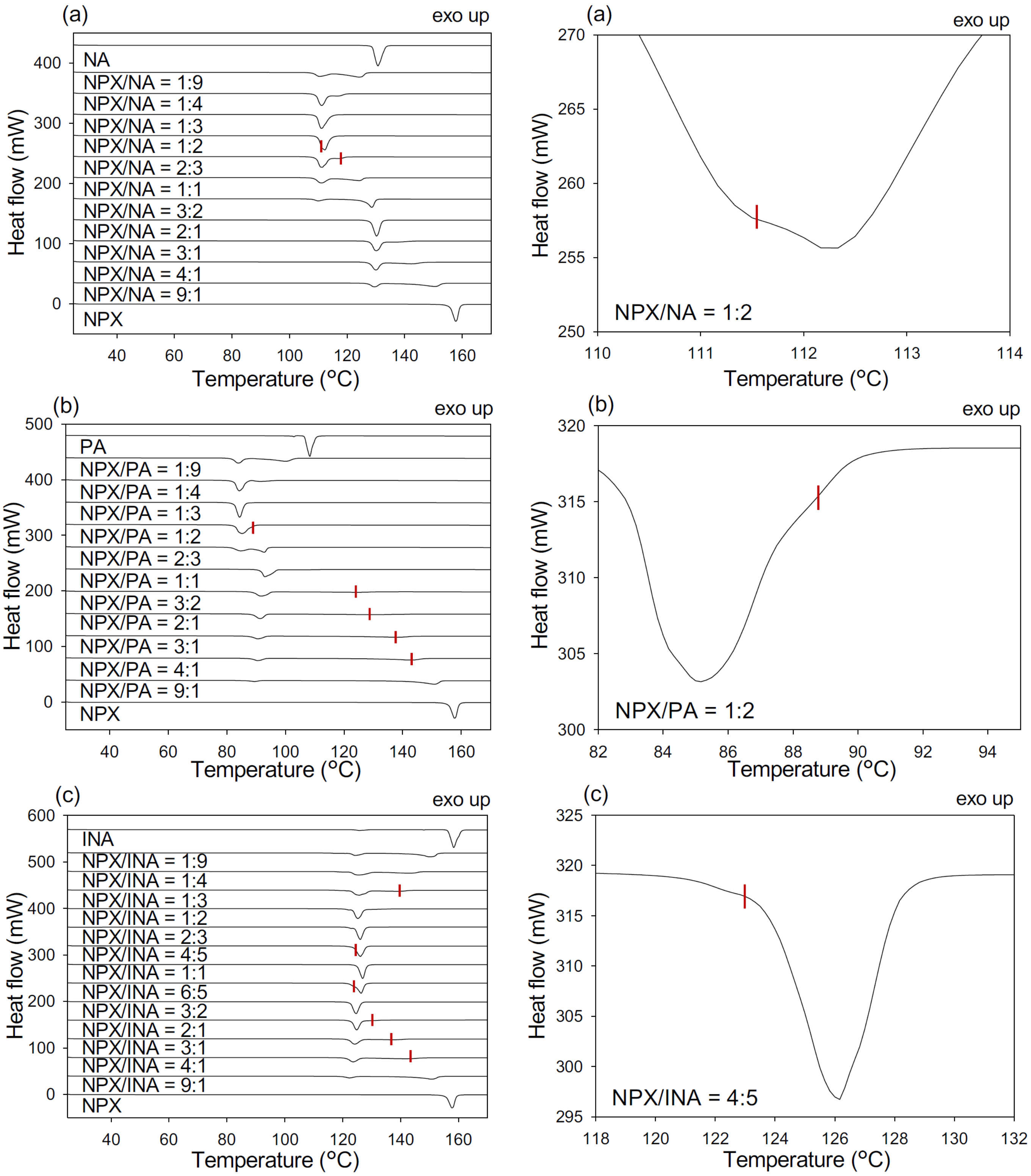

Figure S1. DSC thermograms of (**a**) NPX/NA, (**b**) NPX/PA, and (**c**) NPX/INA mixtures.

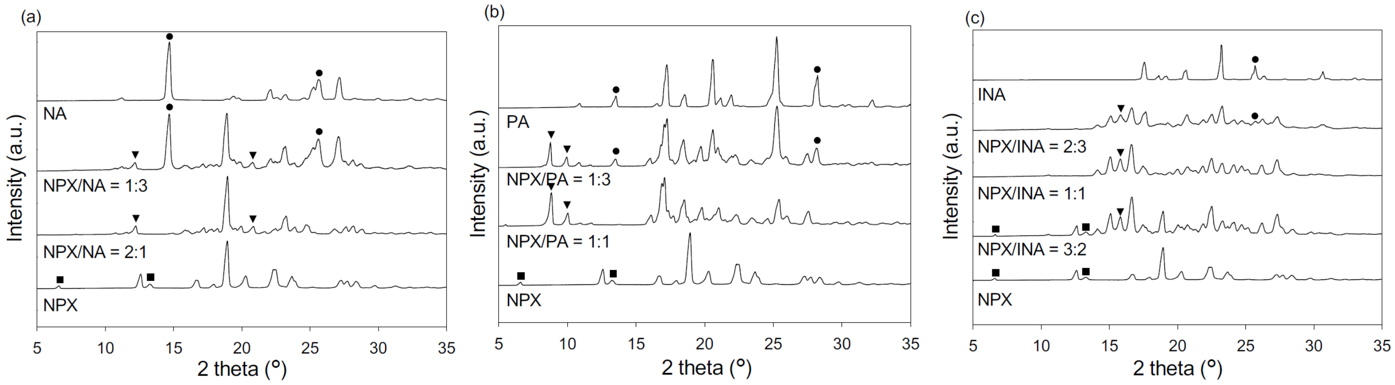

Figure S2. XRD patterns of some mixtures of (**a**) NPX/NA, (**b**) NPX/PA, and (**c**) NPX/INA.

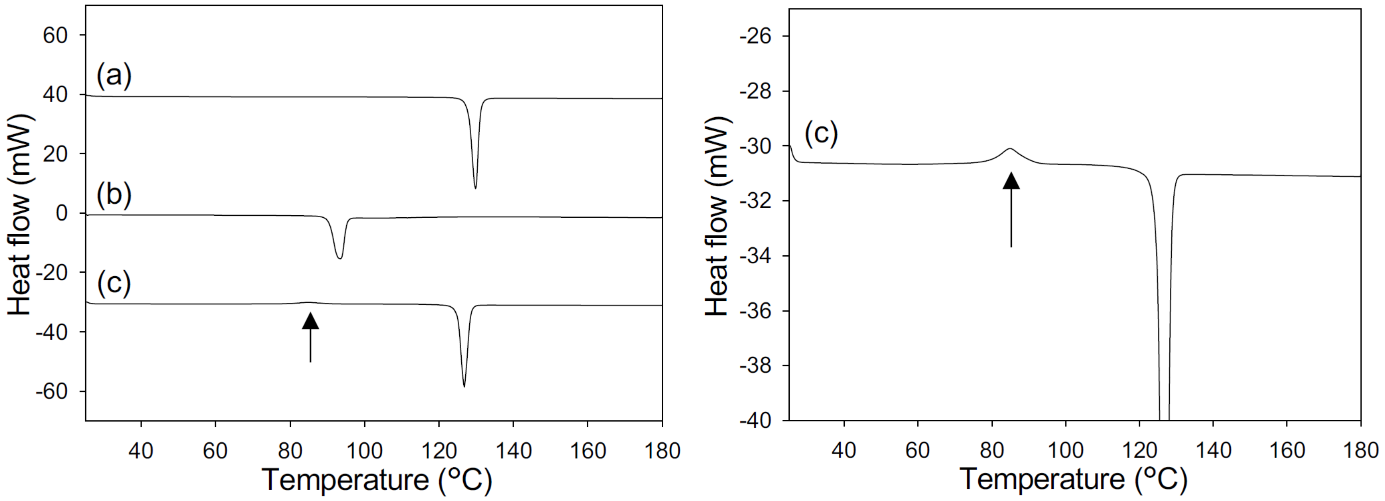

Figure S3. DSC thermograms of melt crystallized cocrystals of (**a**) NPX/NA, (**b**) NPX/PA, and (**c**) NPX/INA.

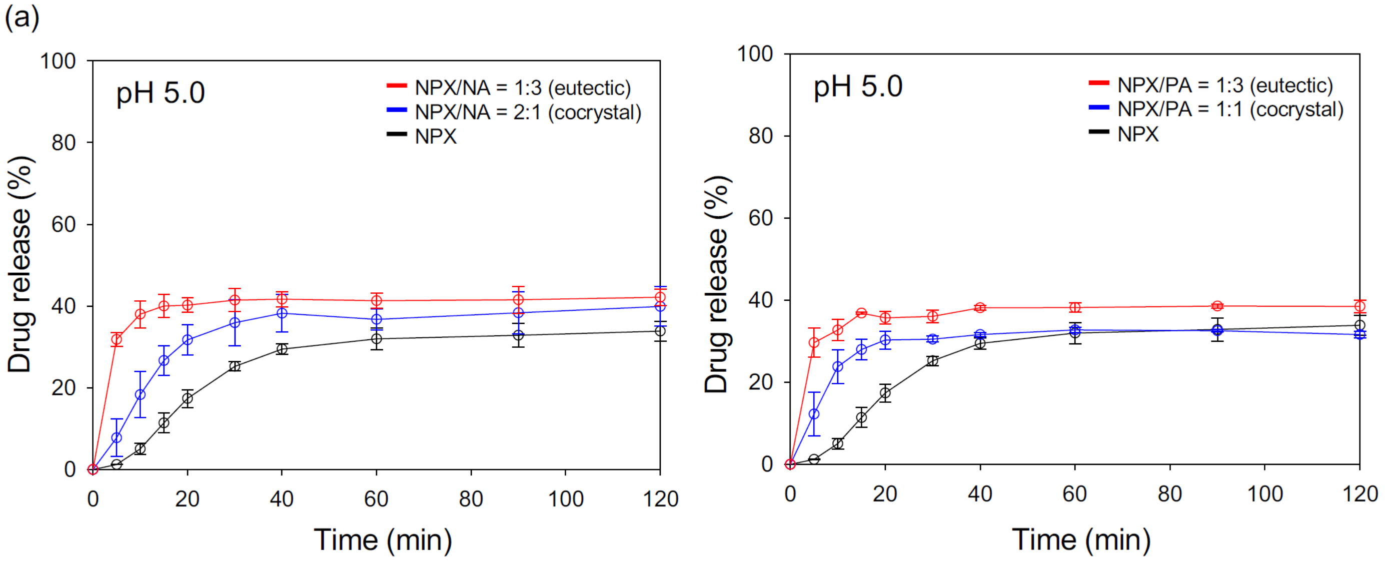

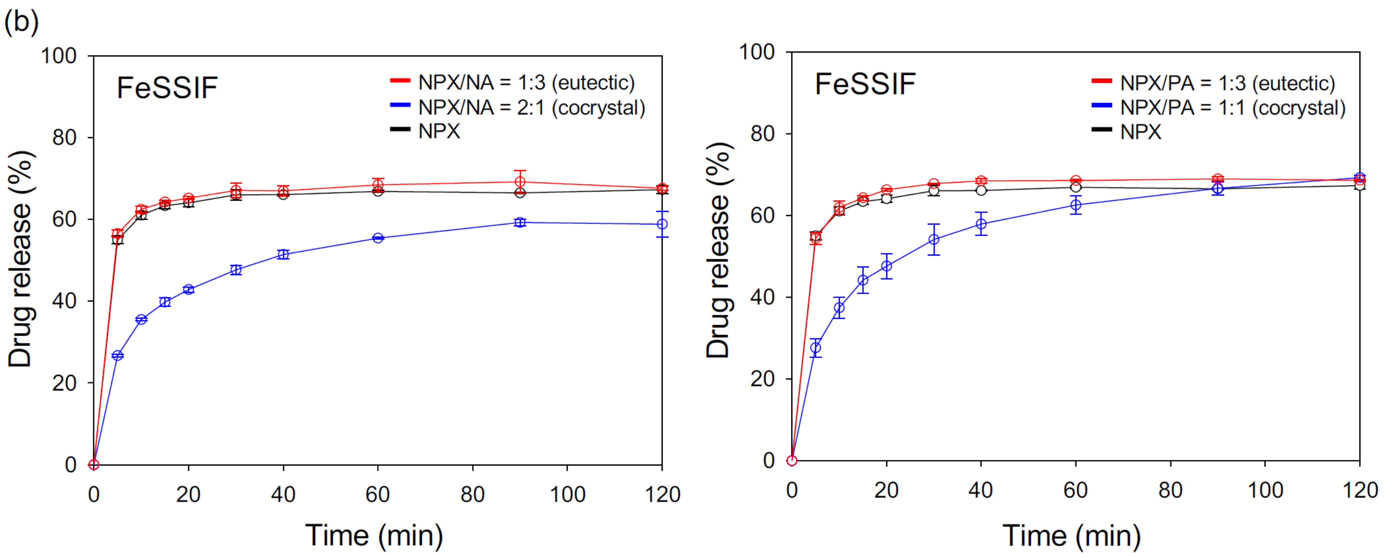

Figure S4. Full dissolution profiles (120 min, n = 3) of NPX/NA and NPX/PA in (**a**) pH 5.0 buffer and (**b**) FeSSIF.
